# Supplementary figures and images for: Multi-Omics Analysis of a Chromosome Segment Substitution Line Reveals a New Regulation Network for Soybean Seed Storage Profile
Source: Int J Mol Sci. 2024 May 21;25(11):5614. doi: 10.3390/ijms25115614 (PMC11171932; doi:10.3390/ijms25115614)

A)

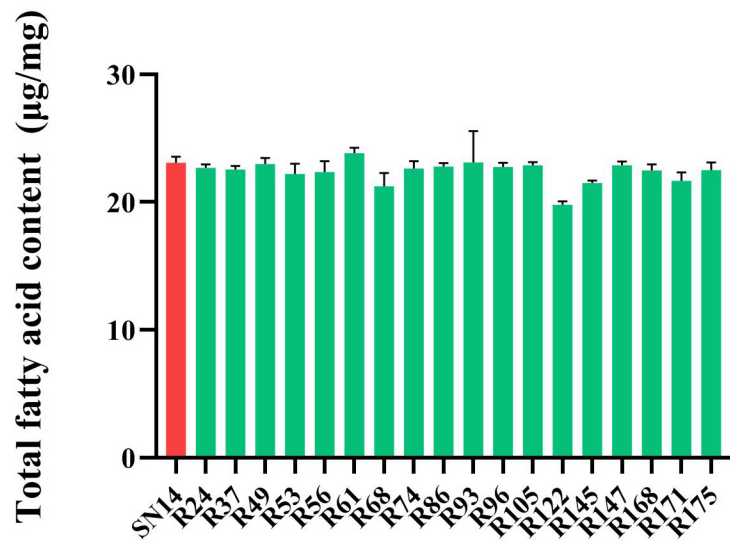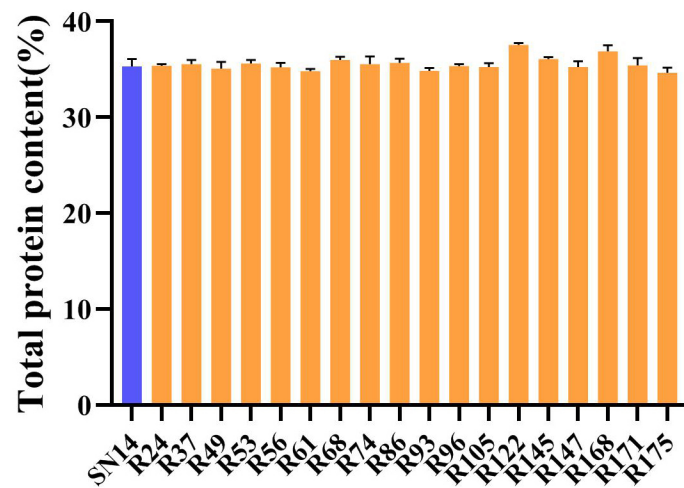

B)

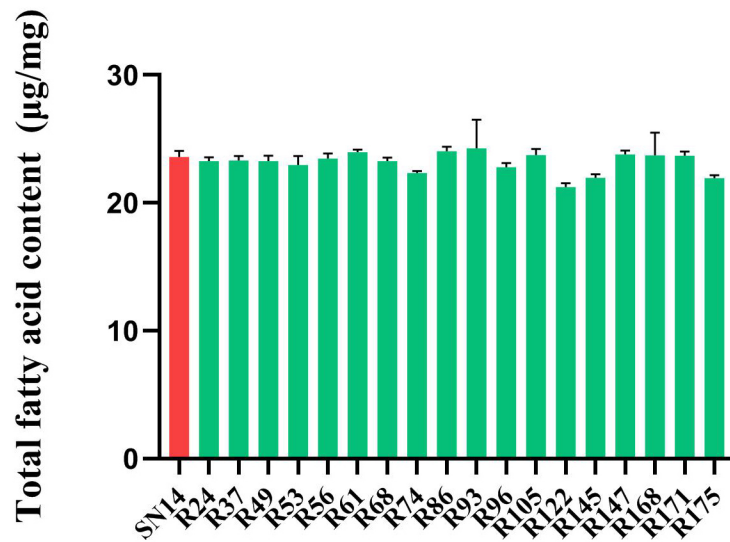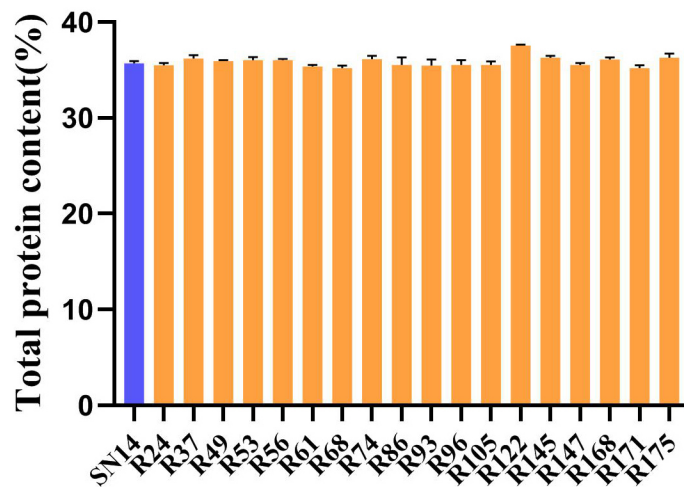

Supplement: Supplementary file 1 [file ijms-25-05614-s001.zip › Figure S1. Total fatty acid content and protein content of screened CSSL population.pdf]

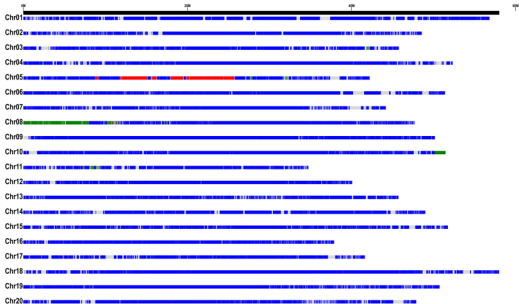

Supplement: Supplementary file 1 [file ijms-25-05614-s001.zip › Figure S2. Genome overview of the substituted region in R122.pdf]

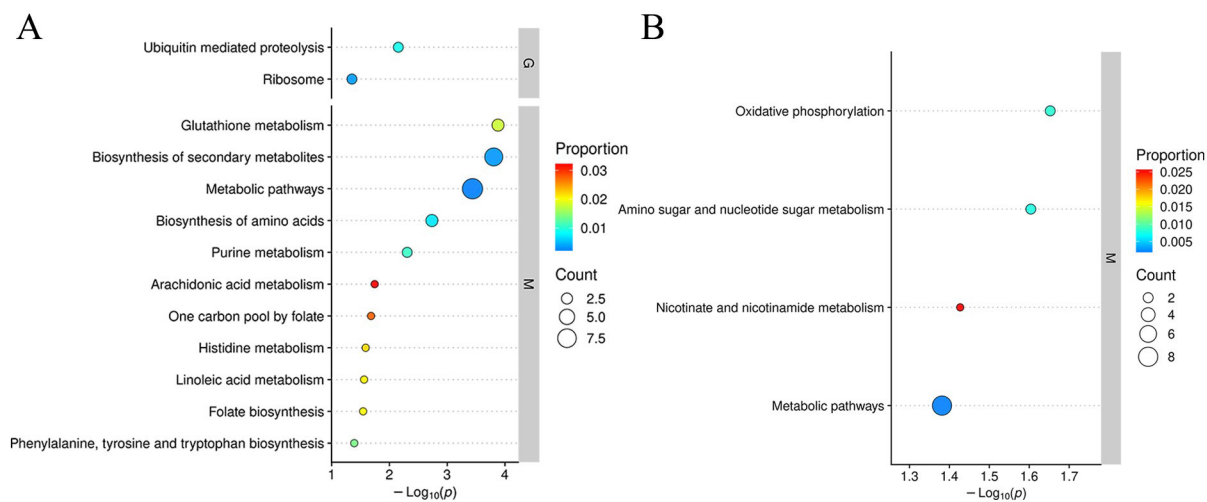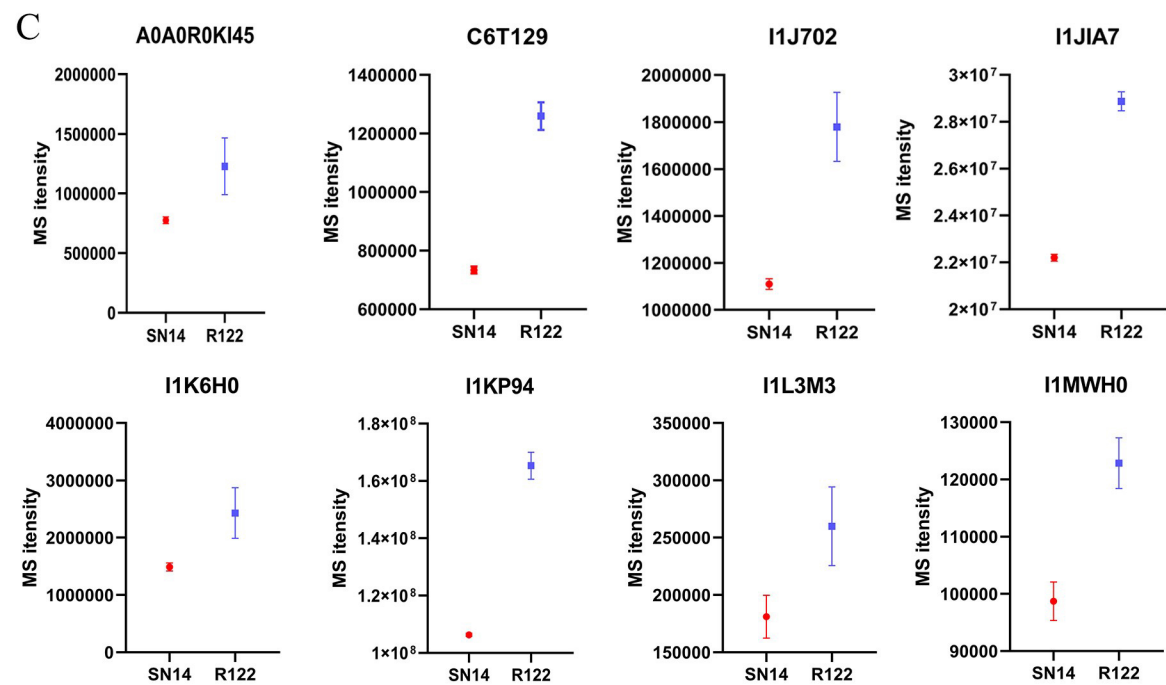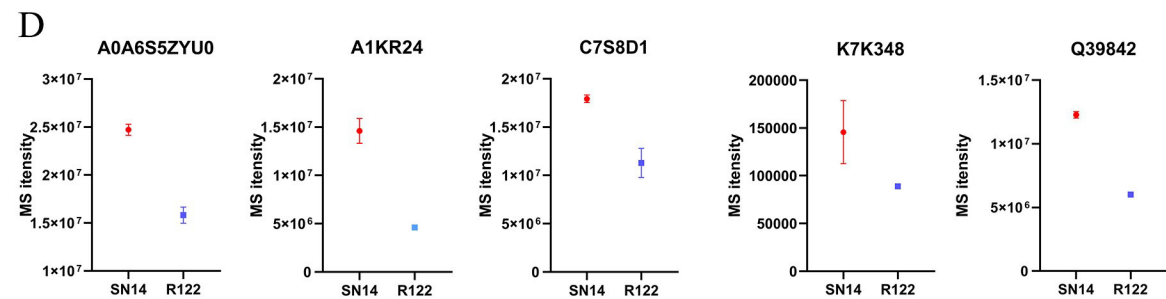

Supplement: Supplementary file 1 [file ijms-25-05614-s001.zip › Figure S3. DEPs with highest and lowest abundance from TMT- based of quantitative proteomics analysis.pdf]

*Glyma.08G137000*

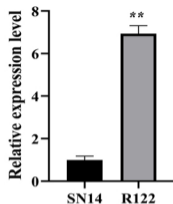

*Glyma.11G017900*

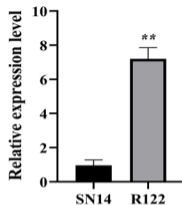

*Glyma.03G068100*

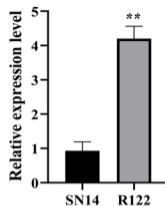

*Glyma.08G286700*

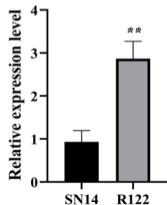

*Glyma.08G279900*

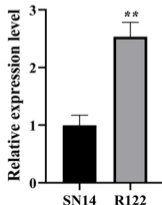

*Glyma.03G036700*

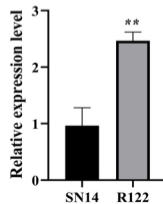

Supplement: Supplementary file 1 [file ijms-25-05614-s001.zip › Figure S4. Relative expression levels of DEGs related to oil and protein acuumulation.pdf]
